# Combined Pharmacologic and Nutritional Modulation of High-Fat Diet-Associated Tumor-Supportive Features in Prostate Cancer Models

**DOI:** 10.3390/biom16070969

**Published:** 2026-07-01

**Authors:** Ke Wu, Qiongyu Hao, Joshua Yang, Yahya Elshimali, Clara E. Magyar, Susanne M. Henning, Ali Andalibi, Piwen Wang

**Affiliations:** 1 Division of Cancer Research and Training, Institute for Advanced Biomedical Research, Charles R. Drew University of Medicine and Science, Los Angeles, CA 90059, USA; 2 Department of Pathology, David Geffen School of Medicine, University of California, Los Angeles, CA 90095, USA; 3 Center for Human Nutrition, David Geffen School of Medicine, University of California, Los Angeles, CA 90095, USA

**Keywords:** prostate cancer, obesity, metabolic dysregulation, inflammation, adipocytes, tumor microenvironment, multi-target therapy, angiogenesis

## Abstract

**Background:** Obesity is associated with aggressive prostate cancer, but the links between metabolic dysregulation, inflammation, adipocyte-associated signaling, and tumor growth remain incompletely defined. This study examined whether high-fat diet (HFD)-associated systemic changes and adipocyte-derived paracrine interactions are linked to prostate cancer growth in preclinical models. **Methods:** An HFD xenograft model and adipocyte co-culture systems were used to evaluate systemic and local tumor-supportive features. Pharmacologic/nutritional modulation was tested using green tea or EGCG, arctigenin, and the CCR2 antagonist RS 504393, alone or in combination. Tumor growth, cell proliferation, angiogenesis-related features, circulating metabolic and cytokine levels, and selected tumor-associated signaling proteins were analyzed. **Results:** HFD feeding was associated with increased circulating free fatty acids, IGF-1, MCP-1, IL-6, and VEGF, together with increased tumor growth, Ki67 staining, and CD31-positive microvessel density. Adipocyte co-culture systems were used to evaluate treatment-associated changes in prostate cancer cell proliferation under adipocyte-associated conditions. Combined modulation with green tea/EGCG, arctigenin, and RS 504393 was associated with greater reductions in adipocyte-associated proliferation, tumor growth, Ki67 staining, and CD31-positive microvessel density than single or dual interventions. Antibody array analysis showed treatment-associated changes in selected stress- and apoptosis-related proteins, including cleaved caspase-7 and phosphorylated Chk1. **Conclusions:** HFD-associated metabolic and inflammatory alterations, adipocyte-associated interactions, proliferative activity, angiogenesis-related features, and stress/apoptosis-related signaling changes were linked within a tumor-supportive framework in preclinical prostate cancer models. Combined pharmacologic/nutritional modulation was associated with reduced tumor-supportive features under HFD conditions. Further mechanistic and translational validation is needed.

## 1. Introduction

Prostate cancer (PCa) remains the most frequently diagnosed malignancy among men worldwide and continues to represent a leading cause of cancer-related mortality [1,2,3,4]. While androgen receptor (AR) signaling is recognized as a central driver of prostate tumor initiation and progression, clinical outcomes vary widely among patients, suggesting that tumor-intrinsic signaling alone does not fully account for disease aggressiveness [5,6,7,8,9]. Increasing epidemiological evidence shows that obesity is associated with a higher incidence of aggressive PCa, increased biochemical recurrence, and elevated prostate cancer-specific mortality [10,11,12,13,14]. These findings suggest that host metabolic status may influence tumor behavior rather than serving only as a background condition [9,13,15]. However, the mechanisms linking obesity-associated metabolic alterations to prostate cancer progression remain incompletely understood [9,11,14].

Obesity is characterized by systemic metabolic dysregulation together with chronic low-grade inflammation [11,12,13,14,16]. Elevated circulating free fatty acids (FFAs), increased insulin-like growth factor-1 (IGF-1), altered adipokine secretion, and sustained inflammatory activation are key features of this state [15,17]. These changes tend to occur in parallel rather than as isolated events and may affect multiple biological processes [15,18]. Importantly, many of these systemic signals intersect with pathways that are known to regulate prostate cancer biology [8,9,14,19]. IGF-1 can activate the PI3K/AKT signaling cascade, can promote cellular proliferation and survival, and has been reported to interact with AR signaling [14,20]. Because AR remains a dominant transcriptional regulator in prostate cancer, obesity-associated endocrine and metabolic signals may influence AR-related biology and tumor behavior [6]. Nevertheless, direct regulation of AR activity by obesity-associated signals requires experimental validation in specific model systems. A key question, therefore, is whether systemic endocrine and metabolic alterations associated with obesity are linked to enhanced prostate tumor growth in vivo [15].

In addition to endocrine signaling, lipid overflow represents another important feature of obesity. Increased FFAs may affect membrane composition, intracellular redox balance, metabolic adaptation, and receptor-associated signaling, thereby influencing growth-related pathways [16,21]. In prostate cancer cells, metabolic reprogramming is closely linked to androgen signaling and cellular adaptation [17]. Thus, obesity-related metabolic stress may not simply provide excess nutrients but may also be associated with altered signaling networks that support tumor expansion. Whether systemic metabolic activation is accompanied by enhanced tumor growth in vivo remains an important issue.

Local adipose tissue may also contribute to tumor biology [22,23,24]. The prostate is surrounded by periprostatic adipose tissue, which functions not only as structural support but also as an active endocrine and paracrine organ. Adipocytes release FFAs, IGF-1, monocyte chemoattractant protein-1 (MCP-1), and other inflammatory mediators that may influence adjacent tumor cells [25]. Bidirectional communication between adipocytes and prostate cancer cells may reinforce proliferative signaling, support survival pathways, and contribute to a microenvironment that favors tumor growth [9,18]. However, direct experimental evidence for adipocyte-associated effects on prostate cancer cell proliferation remains limited. Further evaluation of adipocyte–tumor interactions is therefore needed to clarify the potential role of the local adipose microenvironment in PCa progression.

Chronic inflammation is another defining feature of obesity. Elevated MCP-1 levels are associated with immune cell recruitment, stromal activation, and cytokine production within the tumor microenvironment. In parallel, inflammatory signaling is often associated with increased expression of vascular endothelial growth factor (VEGF), which supports angiogenesis required for sustained tumor growth [26,27]. In prostate cancer, tumor expansion depends on both cellular proliferation and adequate vascular support, suggesting that inflammatory–angiogenic signaling may contribute to disease progression [28]. Notably, metabolic and inflammatory processes are closely interconnected. Lipid accumulation can enhance inflammatory responses, while inflammatory mediators can further promote angiogenesis and tumor growth [29,30]. Together, these processes may create tumor-supportive conditions under obesogenic states.

Despite growing recognition of this network-like behavior, many experimental and therapeutic approaches focus on individual signaling pathways. However, in the context of obesity, parallel metabolic, endocrine, inflammatory, and adipocyte-derived signals may interact or compensate for one another. As a result, modulation of a single pathway may be insufficient to fully suppress tumor-supportive biology [31,32]. Whether combined pharmacologic or nutritional modulation of multiple obesity-associated signaling processes can more effectively reduce prostate tumor growth remains an open question.

To address these issues, we used complementary HFD xenograft and adipocyte co-culture models to examine whether diet-associated systemic metabolic/inflammatory alterations and adipocyte-associated paracrine conditions are linked to prostate cancer growth, and whether combined pharmacologic/nutritional modulation can reduce these tumor-supportive features. Green tea/EGCG, arctigenin, and CCR2 blockade were selected based on their reported effects on prostate cancer signaling, metabolic regulation, inflammatory pathways, and the tumor microenvironment [33,34,35,36,37]. Because these agents may exert pleiotropic biological effects, they were not considered strictly selective inhibitors of single pathways. In the HFD xenograft model, HFD feeding was associated with increased circulating metabolic and inflammatory mediators, enhanced tumor growth, increased Ki67 staining, greater CD31-positive microvessel density, and treatment-associated changes in selected stress/apoptosis-related signaling proteins. In the adipocyte co-culture system, we evaluated how EGCG, arctigenin, RS 504393, and their combinations affected prostate cancer cell proliferation under adipocyte-associated conditions. Overall, these findings support a preclinical working model in which HFD-associated systemic changes, adipocyte-associated interactions, proliferative activity, angiogenesis-related features, and stress/apoptosis-related signaling changes are linked within a tumor-supportive framework, and in which combined pharmacologic/nutritional modulation is associated with reduced tumor-supportive features.

## 2. Materials and Methods

### 2.1. Cell Lines and Reagents

Androgen-sensitive human prostate cancer cell lines LNCaP and LAPC-4 were obtained from the American Type Culture Collection (ATCC, Manassas, VA, USA). Normal human prostate epithelial cells (PrECs) were purchased from Lonza (Walkersville, MD, USA). The murine preadipocyte cell line 3T3-L1 was obtained from ATCC (Manassas, VA, USA). Epigallocatechin-3-gallate (EGCG) was purchased from Sigma-Aldrich (St. Louis, MO, USA). Tea bags were purchased from Celestial Seasonings (Boulder, CO, USA). Arctigenin (Arc, purity ≥ 98%) was obtained from Bolise Co., Ltd. (Xiamen, China). RS 504393, a CCR2 antagonist, was purchased from Tocris Bioscience (Minneapolis, MN, USA). Antibodies against Ki67 and CD31 were purchased from DAKO North America Inc. (Carpinteria, CA, USA).

All reagents were prepared according to the manufacturer’s instructions. Stock solutions for in vitro experiments were dissolved in dimethyl sulfoxide (DMSO; Sigma-Aldrich) and diluted in culture medium to the indicated final working concentrations. The final concentration of DMSO was kept consistent across treatment and control conditions.

### 2.2. Cell Culture

LNCaP and LAPC-4 cells were maintained in RPMI-1640 medium (Gibco, Thermo Fisher Scientific, Waltham, MA, USA) supplemented with 10% fetal bovine serum (FBS; Gibco) and 1% penicillin–streptomycin (Gibco). PrECs were cultured in prostate epithelial growth medium (Lonza) according to the manufacturer’s instructions. Cells were maintained at 37 °C in a humidified incubator with 5% CO_2_.

### 2.3. 3T3-L1 Adipocyte Differentiation and Co-Culture System

3T3-L1 preadipocytes were cultured in DMEM (Gibco) containing 10% bovine calf serum until confluence. Differentiation was induced using standard adipogenic induction medium containing 0.5 mM isobutylmethylxanthine (IBMX; Sigma-Aldrich), 1 μM dexamethasone (Sigma-Aldrich), and 10 μg/mL insulin (Sigma-Aldrich). After 48 h, cells were maintained in insulin-containing medium for an additional 5–7 days until mature adipocyte-like cells formed.

For co-culture experiments, differentiated 3T3-L1 adipocytes were plated in Transwell inserts (Corning Inc., Corning, NY, USA), and prostate cancer cells were seeded in the lower chambers. This system was used as a simplified adipocyte-based model to examine paracrine interactions between differentiated adipocytes and prostate cancer cells. It was not intended to fully reproduce the complexity of human obese or periprostatic adipose tissue. Cells were treated with EGCG (40 μM), Arc (10 μM), RS 504393 (10 or 20 μM), or their combinations as indicated.

### 2.4. Cell Proliferation Assay

Cells were seeded into opaque-wall 96-well plates at a density of 8 × 10^3^ cells per well in quadruplicate and treated with EGCG, Arc, RS 504393, or their combinations at the indicated concentrations for 48 h. To minimize potential effects of hydrogen peroxide generated by autoxidation and/or dimerization of phytochemicals in the cell culture medium [38], catalase (50 U/mL) was added to the medium before treatment with EGCG, Arc, and RS 504393. Cell proliferation was measured using the Cell Titer-Glo^®^ Luminescent Cell Viability Assay kit (Promega Corporation, Madison, WI, USA), which measures intracellular adenosine triphosphate (ATP) as a surrogate for viable cell numbers. Proliferation rates were normalized to untreated controls.

### 2.5. Preparation of GT, Arc, and RS Solution for Animal Study

Green tea (GT) was prepared by brewing one tea bag in 240 mL of boiling water for 5 min. The brewed GT contained the following catechins (mg/L): EGCG 388 ± 12, EGC 204 ± 4, EC 44 ± 2, ECG 64 ± 7, and catechin 7 ± 1. GT was freshly prepared on Monday, Wednesday, and Friday and provided to mice as drinking water ad libitum throughout the study. Whole GT was used for the in vivo study to approximate a dietary intervention and to reflect exposure to a mixture of green tea catechins, whereas purified EGCG was used in vitro to allow controlled exposure to a defined catechin.

Arc and RS 504393 were dissolved in a vehicle consisting of 2% DMSO in corn oil and administered by daily oral gavage at doses of 50 mg/kg and 5 mg/kg body weight, respectively. The concentrations used in vitro and the doses used in vivo were selected based on prior laboratory experience, published preclinical studies, and tolerability considerations. These doses were intended for experimental evaluation of treatment-associated biological effects in preclinical models and should not be interpreted as directly translatable clinical dosing regimens.

### 2.6. Animal Studies and High-Fat Diet Model

All animal procedures were approved by the Institutional Animal Care and Use Committee (IACUC) and were conducted in accordance with institutional guidelines. Male severe combined immunodeficiency (SCID) mice, 5–7 weeks of age, were obtained from Charles River Laboratories. Mice were housed under standard laboratory conditions with free access to food and water.

To establish a diet-associated metabolic and inflammatory xenograft model, mice assigned to the high-fat diet (HFD) groups were maintained on a diet containing 45% kcal from fat throughout the study. A parallel low-fat diet (LFD) control group was included to provide a reference for the diet-associated metabolic phenotype. One week after diet initiation, 5 × 10^5^ LAPC-4 cells suspended in Matrigel were subcutaneously injected into the flank of each mouse. Tumor formation was monitored regularly. When tumors reached approximately 10 mm^3^, mice were randomly assigned to the following groups: LFD control, HFD control, HFD + GT, HFD + Arc, HFD + GT + Arc, HFD + RS 504393, HFD + GT + Arc + RS 504393, and HFD + enzalutamide. Randomization was performed before treatment initiation to minimize allocation bias.

GT, Arc, and RS 504393 were prepared and administered as described in Section 2.5. The HFD + enzalutamide group was included as a pharmacologic comparator for androgen receptor pathway inhibition in androgen-responsive LAPC-4 xenografts. This group was not considered part of the metabolic/inflammatory pharmacologic modulation strategy involving GT, Arc, and RS 504393. Enzalutamide was prepared in a vehicle consisting of 2% DMSO in corn oil and administered by oral gavage at 10 mg/kg body weight daily for 6 weeks.

Mice were monitored throughout the study for general appearance, behavior, mobility, grooming, food and fluid intake, and signs of treatment-related intolerance. Body weight was recorded weekly. Tumor volume was measured weekly using digital calipers and calculated using the formula: length × width × height × 0.5236. Tumor measurements were performed by investigators blinded to treatment allocation whenever feasible.

Predefined exclusion criteria included failure of tumor establishment, severe illness unrelated to tumor burden, unexpected death before treatment initiation, or excessive body weight loss requiring euthanasia according to institutional animal welfare guidelines. No animals were excluded after randomization unless they met these predefined criteria. Treatments were continued for 6 weeks. At the study endpoint, mice were euthanized, and tumors were excised, weighed, and processed for serum analysis, immunohistochemistry, antibody array analysis, and other molecular assays.

### 2.7. Serum Metabolic and Cytokine Analysis

Blood samples were collected by cardiac puncture at the study endpoint. Serum was separated by centrifugation. Free fatty acids (FFAs) and triglycerides were measured using colorimetric assay kits (Sigma-Aldrich). Serum IGF-1, MCP-1, IL-6, and VEGF levels were quantified using ELISA kits (R&D Systems, Minneapolis, MN, USA) according to manufacturer protocols.

### 2.8. Immunohistochemistry (IHC)

Tumor tissues were fixed in 10% formalin (Thermo Fisher Scientific), embedded in paraffin, and assembled for tissue microarray and immunohistochemical detection as described previously [36]. Tissue microarray cores were stained with monoclonal antibodies against Ki67 and CD31 to assess tumor cell proliferation and microvessel density, respectively. After deparaffinization and antigen retrieval, slides were incubated with the primary antibodies, followed by appropriate secondary detection reagents and hematoxylin counterstaining. Slides were digitized, and morphometric analysis was performed using Definiens Tissue Studio software version 4.0 (Definiens AG, Munich, Germany).

For quantitative analysis, five tumors from each treatment group were evaluated as independent biological replicates. For each tumor, a total of 6 cylindrical cores 1.0 mm in diameter were transferred to construct tissue microarrays. Necrotic areas, tissue folds, edge artifacts, and areas with poor staining quality were excluded from analysis. Ki67 staining was quantified as the percentage of Ki67-positive tumor nuclei among total tumor nuclei within the analyzed tumor area. CD31 staining was quantified as microvessel density, expressed as the number of CD31-positive vessels per field. Quantification was performed by investigators blinded to treatment allocation. The average value from the analyzed fields was calculated for each tumor, and each tumor, rather than each field, was treated as an independent biological replicate for statistical analysis.

### 2.9. Antibody Array Analysis of Tumor Signaling Proteins

To further evaluate treatment-associated molecular changes in tumor tissues, antibody array analysis was performed using tumor lysates from the in vivo study. Five tumor samples were randomly selected from each treatment group. Total protein was extracted from frozen tumor tissues using RIPA lysis buffer supplemented with protease and phosphatase inhibitors. Protein concentrations were determined and adjusted to 0.5 mg/mL using the array diluent buffer provided by the manufacturer.

A slide-based antibody array was performed using the PathScan Stress and Apoptosis Signaling Antibody Array Kit (Cell Signaling Technology, Danvers, MA, USA) according to the manufacturer’s protocol. This platform allows simultaneous detection of 19 signaling molecules involved in stress response, DNA damage signaling, and apoptosis. Briefly, diluted tumor protein lysates were incubated with array slides overnight at 4 °C. After washing, slides were incubated with the detection antibody cocktail, followed by HRP-linked streptavidin and chemiluminescent substrate. Signals were visualized using a ChemiDoc XRS imaging system. Each target protein was spotted in duplicate on the array. Spot intensities were quantified using image analysis software and normalized to the corresponding internal loading control. Data are presented as mean ± SD. Cleaved caspase-7 and phosphorylated Chk1 were selected for focused quantitative comparison based on visible treatment-associated changes observed in the antibody array screening and their biological relevance to apoptosis and stress/DNA damage signaling.

### 2.10. Statistical Analysis

Data are presented as mean ± standard deviation (SD) unless otherwise indicated. Statistical analyses were performed using GraphPad Prism software version 9.0 (GraphPad Software, San Diego, CA, USA). For endpoint measurements, including serum metabolic and cytokine levels, final tumor weight, body weight, immunohistochemical quantification, and antibody array signal intensity, comparisons among multiple groups were performed using one-way ANOVA followed by Tukey’s post hoc test. Tumor growth curves over time were analyzed using two-way repeated-measures ANOVA followed by multiple-comparison testing where appropriate.

For in vivo treatment analyses, HFD control was used as the primary reference group unless otherwise specified. Comparisons between LFD control and HFD control were used to evaluate the diet-associated metabolic and tumor-growth phenotype. The HFD + enzalutamide group was included as a pharmacologic comparator for androgen receptor pathway inhibition and was not considered part of the metabolic/inflammatory combination strategy. Therefore, the primary comparisons for the metabolic/inflammatory intervention were made between HFD control and the GT, Arc, GT + Arc, RS 504393, and GT + Arc + RS groups.

For in vitro experiments, data were obtained from at least three independent experiments, with technical replicates included as indicated in the figure legends. A *p* value < 0.05 was considered statistically significant.

## 3. Results

### 3.1. High-Fat Diet Is Associated with Systemic Metabolic and Inflammatory Alterations and Enhanced Tumor Growth

To examine the relationship between diet-associated metabolic alterations and prostate cancer growth, we established an HFD xenograft model using LAPC-4 cells. A parallel LFD control group was included to provide a reference for the diet-associated metabolic phenotype. During the treatment period, mouse body weight was monitored weekly to assess general metabolic changes and treatment tolerability. HFD-fed mice showed an increased body weight trend compared with LFD control mice, consistent with diet-associated metabolic alterations (Supplementary Figure S1).

**Figure 1:**
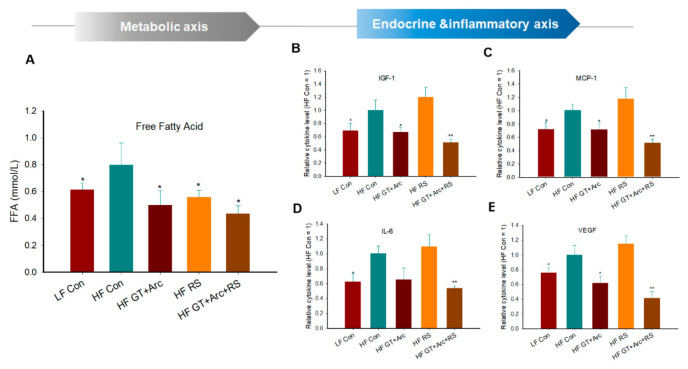
Serum metabolic and inflammatory markers in LAPC-4 tumor-bearing mice maintained under low-fat diet or high-fat diet conditions. (**A**) Serum free fatty acid (FFA) levels. (**B**) Serum IGF-1 levels. (**C**) Serum MCP-1 levels. (**D**) Serum IL-6 levels. (**E**) Serum VEGF levels. Groups included low-fat diet control (LF Con), high-fat diet control (HF Con), and HFD-fed mice treated with green tea (GT), arctigenin (Arc), GT + Arc, RS 504393 (RS), or GT + Arc + RS, as indicated. Data are presented as mean ± SD (*n* = 10 mice per group). Comparisons were performed using one-way ANOVA followed by Tukey’s post hoc test. * *p* < 0.05 and ** *p* < 0.01 versus HF Con, unless otherwise indicated. FFA, free fatty acid; IGF-1, insulin-like growth factor-1; MCP-1, monocyte chemoattractant protein-1; IL-6, interleukin-6; VEGF, vascular endothelial growth factor.

Serum analysis showed that HFD feeding was associated with higher circulating levels of free fatty acids (FFAs) (*p* = 0.04), IGF-1 (*p* = 0.02), MCP-1 (*p* = 0.03), IL-6 (*p* = 0.02), and VEGF (*p* = 0.03), by comparing HFD control with LFD control mice (Figure 1). These systemic changes were accompanied by increased tumor growth under HFD conditions, supporting an association between diet-associated metabolic/inflammatory alterations and enhanced tumor growth in this xenograft model.

During the treatment period, no excessive body weight loss was observed in the GT + Arc, RS 504393, or GT + Arc + RS 504393 treatment groups compared with the HFD control group (Supplementary Figure S1). In addition, routine animal monitoring did not reveal severe treatment-related adverse signs requiring early euthanasia under the experimental conditions used. These observations support the general tolerability of the in vivo treatment regimens.

### 3.2. Adipocyte Co-Culture System Shows Treatment-Associated Changes in Prostate Cancer Cell Proliferation

**Figure 2:**
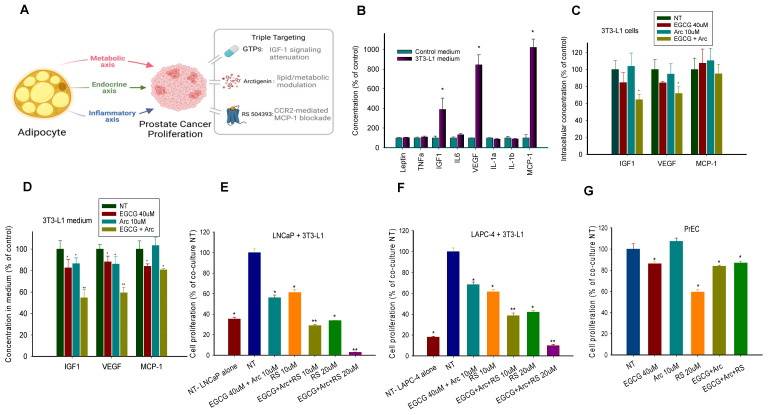
Adipocyte co-culture system and treatment-associated changes in prostate cancer cell proliferation. (**A**) Schematic illustration of the adipocyte–prostate cancer cell co-culture system and the pharmacologic/nutritional modulators examined in this study. The schematic summarizes the experimental framework used to evaluate adipocyte-associated paracrine interactions and combined modulation with EGCG, arctigenin (Arc), and RS 504393 (RS). (**B**) Previously published contextual data adapted from Hao et al. [39]. This panel shows cytokine and growth factor levels in differentiated 3T3-L1 adipocyte-conditioned medium compared with blank complete medium, including IGF-1, VEGF, MCP-1, and IL-6. This panel is included only to provide biological context for the adipocyte-conditioned medium and co-culture system. It is not presented as newly generated data from the current study. (**C**) Intracellular levels of IGF-1, VEGF, and MCP-1 in differentiated 3T3-L1 adipocytes treated with EGCG, Arc, or EGCG + Arc. (**D**) Levels of IGF-1, VEGF, and MCP-1 in conditioned medium from differentiated 3T3-L1 adipocytes treated with EGCG, Arc, or EGCG + Arc. (**E**) Proliferation of LNCaP cells cultured alone or co-cultured with differentiated 3T3-L1 adipocytes and treated with RS 504393, EGCG + Arc, or the indicated combined treatments. Proliferation was normalized to untreated co-culture controls (NT). (**F**) Proliferation of LAPC-4 cells under the same co-culture and treatment conditions. (**G**) Proliferation of normal prostate epithelial cells (PrECs) treated with EGCG, Arc, RS 504393, or their combinations. Data are presented as mean ± SD from three independent experiments with at least four technical replicates per treatment condition. Statistical comparisons were performed as indicated in the figure. * *p* < 0.05 versus NT; ** *p* < 0.01 versus the indicated individual treatment. EGCG, epigallocatechin-3-gallate; Arc, arctigenin; RS, RS 504393; NT, untreated control; PrEC, prostate epithelial cells; IGF-1, insulin-like growth factor-1; VEGF, vascular endothelial growth factor; MCP-1, monocyte chemoattractant protein-1.

To examine prostate cancer cell proliferation under adipocyte-associated conditions, we established co-culture systems using differentiated 3T3-L1 adipocytes and prostate cancer cell lines. This system was primarily used to evaluate treatment-associated changes in prostate cancer cell proliferation in the presence of differentiated adipocytes (Figure 2), rather than to establish a direct quantitative comparison between monoculture and co-culture conditions.

Figure 2B contains previously published contextual data adapted from Hao et al. [39]. This panel shows that differentiated 3T3-L1 adipocyte-conditioned medium contains increased levels of selected cytokines and growth factors, including IGF-1, VEGF, and MCP-1, compared with blank complete medium. This information is included to provide biological context for the adipocyte-conditioned medium and co-culture system and is not presented as newly generated evidence in the current study.

In the current experiments, EGCG and Arc treatment were associated with changes in intracellular and secreted levels of selected adipocyte-associated factors, including IGF-1, VEGF, and MCP-1 (Figure 2C,D). Co-culture with 3T3-L1 adipocytes significantly increased the proliferation of both LNCaP and LAPC-4 cells, as suggested by comparison of the non-treated (NT) co-culture controls with their corresponding NT monoculture controls (Figure 2E,F). In co-culture experiments, pharmacologic modulation with EGCG, Arc, RS 504393, or their combinations reduced prostate cancer cell proliferation under adipocyte-associated conditions to varying degrees (Figure 2E,F). The combined EGCG + Arc + RS 504393 treatment produced the strongest reduction in proliferation in both LNCaP and LAPC-4 co-culture systems (*p* < 0.001 compared with the corresponding EGCG + Arc or RS alone treatment).

### 3.3. Combined Pharmacologic/Nutritional Modulation Is Associated with Reduced Tumor Growth In Vivo Under HFD Conditions

**Figure 3:**
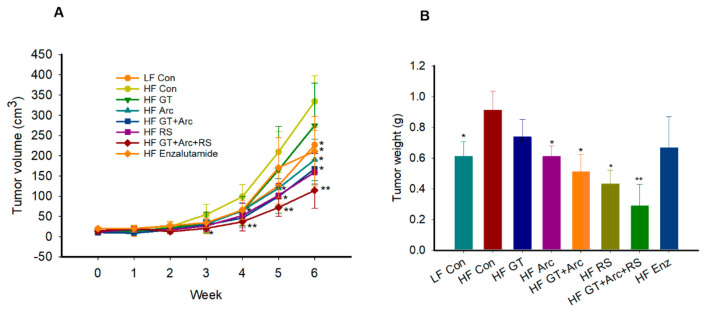
Tumor growth and final tumor weight in LAPC-4 xenograft-bearing mice under low-fat diet or high-fat diet conditions. (**A**) Tumor growth curves of LAPC-4 xenografts in mice fed a low-fat diet (LF Con) or high-fat diet (HF Con), and in HFD-fed mice treated with green tea (GT), arctigenin (Arc), GT + Arc, RS 504393 (RS), GT + Arc + RS, or enzalutamide (Enz). Tumor volumes were measured weekly for 6 weeks using digital calipers. (**B**) Final tumor weights were measured at the study endpoint. The HFD + enzalutamide group was included as a pharmacologic comparator for inhibition of the androgen receptor pathway in androgen-responsive LAPC-4 xenografts and was not considered part of the GT/Arc/RS metabolic/inflammatory modulation strategy. Data are presented as mean ± SD (*n* = 10 mice per group). Tumor growth curves were analyzed using two-way repeated-measures ANOVA followed by multiple-comparison testing, and final tumor weights were analyzed using one-way ANOVA followed by Tukey’s post hoc test. * *p* < 0.05 and ** *p* < 0.01 versus HF Con, unless otherwise indicated. LF, low-fat diet; HF, high-fat diet; HFD, high-fat diet; GT, green tea; Arc, arctigenin; RS, RS 504393; Enz, enzalutamide.

To evaluate whether the treatment patterns observed in vitro were reflected in vivo, we assessed tumor growth in HFD-fed xenograft-bearing mice treated with GT, Arc, GT + Arc, RS 504393, or GT + Arc + RS 504393. Tumor volume and final tumor weight were reduced to varying degrees in the treatment groups compared with HFD control mice (Figure 3A,B). Among the metabolic/inflammatory intervention groups, the combined GT + Arc + RS 504393 regimen showed the most pronounced reduction in tumor growth (*p* = 0.003 versus HFD control at Week 6) and final tumor burden (*p* = 0.008 versus HFD control).

The HFD + enzalutamide group was included as a pharmacologic comparator for inhibition of the androgen receptor pathway in androgen-responsive LAPC-4 xenografts. This group was analyzed and presented separately and was not considered part of the GT/Arc/RS metabolic/inflammatory modulation strategy. These findings support the interpretation that combined pharmacologic/nutritional modulation was associated with reduced tumor growth under HFD conditions, while they do not establish pathway-specific causality.

### 3.4. Combined Pharmacologic/Nutritional Modulation Is Associated with Reduced Ki67 and CD31 Staining in Tumor Tissues

To further evaluate treatment-associated histological changes in tumor tissues, we performed immunohistochemical analyses of Ki67 and CD31. Ki67 staining was used to assess tumor cell proliferative activity, whereas CD31 staining was used to estimate microvessel density.

**Figure 4:**
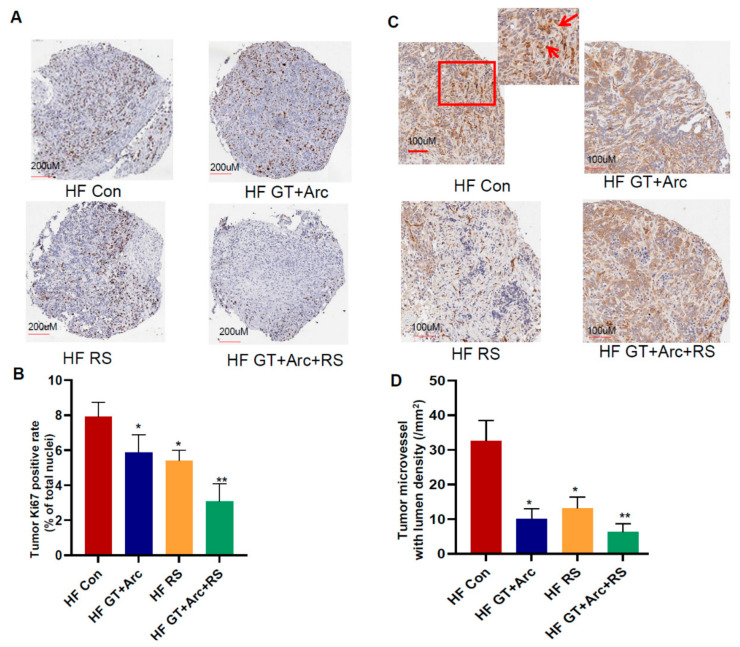
Immunohistochemical analysis of Ki67 and CD31 in LAPC-4 xenograft tumors. (**A**) Representative Ki67-stained tumor sections from HF control and treatment groups. (**B**) Quantification of Ki67 staining, expressed as the percentage of Ki67-positive tumor nuclei among total tumor nuclei within analyzed viable tumor areas. (**C**) Representative CD31-stained tumor sections from HF control and treatment groups. The red box and arrow indicate the representative area/feature highlighted in the image. (**D**) Quantification of CD31 staining, expressed as microvessel density based on the number of CD31-positive vessels per field. Five tumors per group were analyzed as independent biological replicates. For each tumor, representative tumor cores or sections and non-overlapping viable fields were quantified; necrotic areas, tissue folds, edge artifacts, and areas with poor staining quality were excluded. Data are presented as mean ± SEM (*n* = 5 tumors per group). Statistical comparisons were performed using one-way ANOVA followed by Tukey’s post hoc test. * *p* < 0.05 and ** *p* < 0.01 versus HF control, unless otherwise indicated. HF, high-fat diet; GT, green tea; Arc, arctigenin; RS, RS 504393.

Compared with HFD control tumors, treatment groups showed reduced Ki67-positive tumor cell staining, with the strongest reduction observed in the GT + Arc + RS 504393 group (*p* = 0.008) (Figure 4A,B). CD31 staining also showed reduced microvessel density in treated tumors, particularly in the combined treatment group (*p* = 0.001 versus HFD control) (Figure 4C,D). These findings indicate that combined pharmacologic/nutritional modulation was associated with lower tumor cell proliferative activity and reduced angiogenesis-related histological features in this preclinical xenograft model.

### 3.5. Antibody Array Analysis Indicates Treatment-Associated Modulation of Stress- and Apoptosis-Related Signaling Proteins

To further examine whether the in vivo interventions were associated with molecular changes within tumor tissues, we performed antibody array analysis using tumor protein lysates from randomly selected samples in each group. The array simultaneously assessed 19 signaling molecules involved in stress response, DNA damage signaling, and apoptosis.

**Figure 5:**
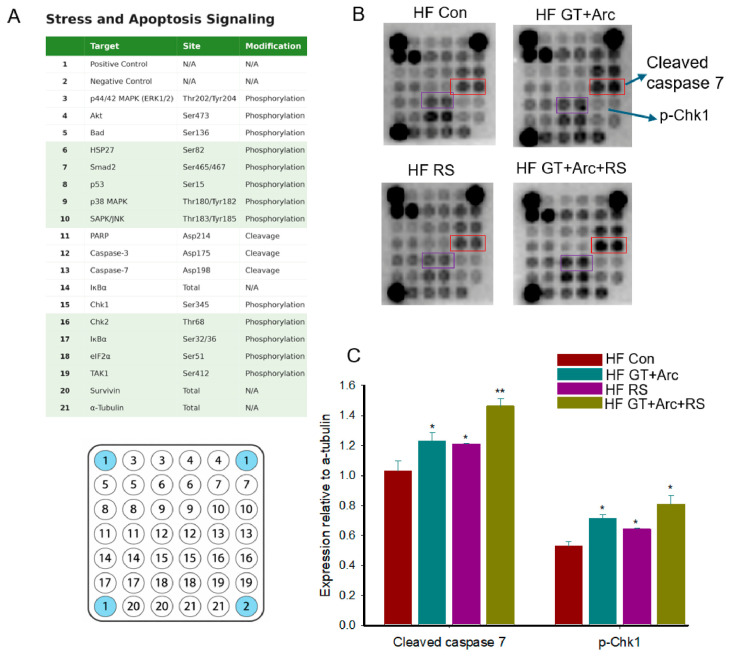
Antibody array analysis of stress- and apoptosis-related signaling proteins in tumor tissues. (**A**) Layout and target map of the PathScan Stress and Apoptosis Signaling Antibody Array used in this study. The array detects 19 selected signaling molecules related to stress response, DNA damage signaling, and apoptosis, together with positive controls, negative controls, and α-tubulin as an internal reference. (**B**) Representative antibody array images from tumor tissues of the HF Con, HF GT + Arc, HF RS, and HF GT + Arc + RS groups. Highlighted spots indicate cleaved caspase-7 and phosphorylated Chk1 signals selected for quantitative comparison. (**C**) Quantification of cleaved caspase-7 and p-Chk1 signal intensities normalized to α-tubulin. Five tumor samples were randomly selected from each group for analysis. Data are presented as mean ± SD. Statistical comparisons were performed using one-way ANOVA followed by Tukey’s post hoc test. * *p* < 0.05 and ** *p* < 0.01 versus HF Con, unless otherwise indicated. HF, high-fat diet; GT, green tea; Arc, arctigenin; RS, RS 504393; p-Chk1, phosphorylated Chk1.

Compared with HFD control tumors, treatment groups showed altered expression or phosphorylation of selected stress- and apoptosis-related proteins. Based on visible treatment-associated changes observed in the antibody array screening and their biological relevance to apoptosis and stress/DNA damage signaling, cleaved caspase-7 and phosphorylated Chk1 were selected for focused quantitative comparison. Both markers showed treatment-associated modulation, with the strongest changes observed in the combined GT + Arc + RS 504393 group (*p* = 0.005 for cleaved caspase-7 and *p* = 0.02 for phosphorylated Chk1 compared with HFD control) (Figure 5). These results suggest that the combined intervention was associated with changes in tumor stress-response and apoptosis-related signaling.

Together with the observed reductions in tumor growth, Ki67 staining, and CD31-positive microvessel density, the antibody array data provide additional molecular context for treatment-associated changes under HFD conditions. However, these findings should be interpreted as evidence of signaling modulation rather than proof of exclusive inhibition of a single pathway or direct validation of IGF-1/PI3K/AKT, AR, VEGFR, CCR2, or NF-κB signaling.

### 3.6. A Working Model Integrates Systemic and Local Features of Obesity-Associated Tumor Support

**Figure 6:**
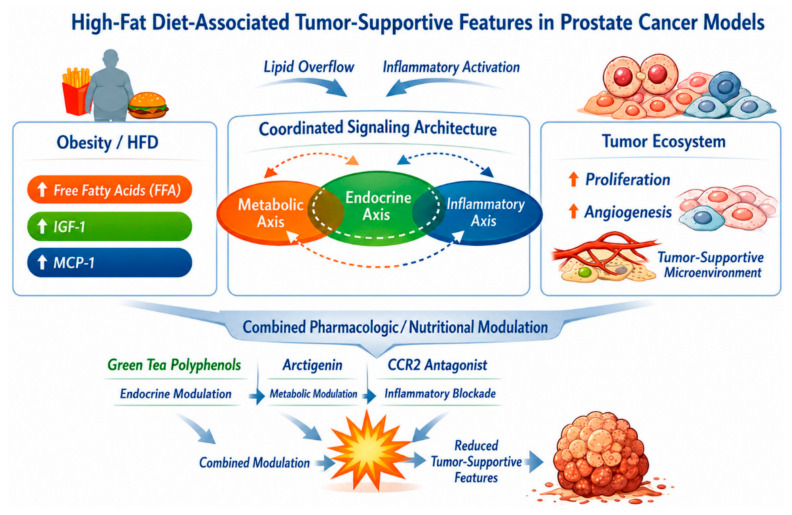
Working model summarizing systemic and local features examined in this study. The schematic integrates HFD-associated systemic metabolic and inflammatory alterations, adipocyte-associated paracrine interactions, tumor growth, proliferative activity, angiogenesis-related changes, and stress/apoptosis-related signaling features observed in the present experimental systems. The intervention-related labels in the schematic are intended to summarize combined pharmacologic/nutritional modulation of tumor-supportive features under HFD-associated conditions and should not be interpreted as evidence of direct intracellular pathway causality, single-molecule dependence, definitive tumor destabilization, or clinical therapeutic efficacy. This schematic is intended as a systems-level working model based on the current in vitro and in vivo observations. HFD, high-fat diet; GT, green tea; Arc, arctigenin; RS, RS 504393.

Based on these findings, we developed a conceptual working model integrating systemic metabolic alterations, adipocyte-associated interactions, tumor growth, angiogenesis, and stress/apoptosis-related signaling changes in obesity-associated prostate cancer (Figure 6). In this model, HFD-associated systemic metabolic and inflammatory alterations may provide tumor-supportive signals that are further influenced by local adipocyte-derived factors. These signals may interact with proliferative, angiogenic, and stress-response processes within the tumor microenvironment.

Combined pharmacologic/nutritional modulation with GT, Arc, and RS 504393 was associated with reduced tumor growth and altered tumor-associated signaling under HFD conditions. This schematic is intended as a systems-level working model based on the present in vitro and in vivo observations. It does not establish single-molecule causality, direct intracellular pathway blockade, or clinical therapeutic efficacy.

## 4. Discussion

In this study, we examined whether HFD-associated systemic metabolic and inflammatory alterations and adipocyte-associated paracrine interactions are linked to prostate cancer growth in complementary preclinical models. Rather than supporting a model in which a single dominant pathway accounts for the observed phenotype, our findings suggest that multiple systemic and local features may act together under HFD conditions. Using an HFD xenograft model and an adipocyte co-culture system, we observed associations among diet-related metabolic and inflammatory changes, adipocyte-associated interactions, tumor growth, proliferative activity, angiogenesis-related features, and selected stress/apoptosis-related signaling changes.

Our in vivo results showed that HFD feeding was associated with increased circulating FFAs, IGF-1, MCP-1, IL-6, and VEGF in tumor-bearing mice, together with increased tumor growth, compared with the LFD control condition. These findings support the concept that host metabolic status may influence tumor behavior rather than functioning only as a background condition. The concurrent elevation of metabolic and inflammatory factors is consistent with prior reports describing interactions between metabolic dysregulation and inflammatory signaling in obesity-associated tumor biology [32,40,41,42]. However, the current HFD xenograft model should be interpreted as a diet-associated metabolic and inflammatory alteration model rather than as a fully characterized metabolic syndrome model.

At the local microenvironmental level, the differentiated 3T3-L1 adipocyte co-culture system was used to evaluate treatment-associated changes in prostate cancer cell proliferation under adipocyte-associated conditions. Pharmacologic modulation using EGCG, arctigenin, RS 504393, or their combinations reduced adipocyte-associated tumor cell proliferation to varying degrees, with the combined treatment showing the strongest reduction in the co-culture assays. Similarly, combined pharmacologic/nutritional modulation with GT, arctigenin, and RS 504393 was associated with reduced tumor growth, decreased Ki67 staining, and lower CD31-positive microvessel density under HFD conditions. These findings suggest treatment-associated changes in tumor growth, proliferative activity, and angiogenesis-related histological features, but they do not establish direct pathway causality.

Several aspects of the pharmacologic interpretation require caution. EGCG, GT, arctigenin, and RS 504393 may have broader biological activities beyond the axes emphasized in this study; therefore, the results should be interpreted as evidence of combined modulation rather than selective pathway inhibition. In addition, whole GT was used in vivo to approximate a dietary intervention containing multiple catechins, whereas purified EGCG was used in vitro to provide controlled exposure to a defined catechin. Because the in vivo design did not include an HFD + purified EGCG-only group, the observed GT-associated effects cannot be attributed specifically to EGCG alone. Future studies directly comparing purified EGCG, whole GT, and dose-matched catechin mixtures in vivo will be needed to define the relative contribution of EGCG and other green tea components. Other diet-derived polyphenol metabolites have also been investigated in preclinical prostate cancer models, including pomegranate ellagitannin-derived metabolites that were reported to inhibit prostate cancer growth and localize to prostate tissue [43].

The antibody array analysis provided additional molecular context by showing treatment-associated modulation of selected stress- and apoptosis-related proteins, including cleaved caspase-7 and phosphorylated Chk1, with the most prominent changes observed in the combined GT + Arc + RS group (Figure 5). These data are consistent with the observed reductions in tumor growth, Ki67 staining, and CD31-positive microvessel density. However, the array provides a targeted survey of selected proteins and does not establish direct pathway causality. The observed changes cannot be attributed exclusively to inhibition of the IGF-1, lipid-metabolic, MCP-1/CCR2, AR, VEGFR, PI3K/AKT, ERK, or NF-κB pathways. Future studies using pathway-specific biochemical assays, genetic approaches, pharmacodynamic analyses, transcriptomics, or proteomics will be required to define the precise molecular mechanisms involved.

Based on these observations, the working model proposed here (Figure 6) integrates HFD-associated systemic metabolic and inflammatory alterations, adipocyte-associated paracrine interactions, tumor growth, proliferation, angiogenesis-related features, and stress/apoptosis-related signaling changes. This model should be interpreted as a systems-level working framework rather than as a validated intracellular pathway map or proof of single-molecule causality. Similar interactions between metabolic dysregulation and inflammation have been reported in other cancers, including breast, colorectal, and pancreatic malignancies [12,15,44], suggesting that this pattern may not be unique to prostate cancer. However, whether similar network-level interactions occur in human obesity-associated prostate cancer will require validation using clinical biospecimens and models that more closely reproduce the human tumor microenvironment.

From a translational perspective, the current findings should be interpreted as preclinical evidence supporting a working biological framework rather than as direct therapeutic evidence. Recent biomarker-oriented work in prostate cancer has emphasized that therapeutic interpretation depends not only on tumor-intrinsic features but also on methodological considerations and the broader tumor microenvironment [45]. The present study does not establish clinical efficacy or therapeutic applicability of the tested interventions.

Several limitations should be considered. First, the study did not include complete metabolic phenotyping, such as body composition, fasting glucose, insulin, HOMA-IR, leptin, adiponectin, or glucose tolerance testing. Second, differentiated 3T3-L1 adipocytes represent a simplified adipocyte-based co-culture system and do not fully reproduce human obese or periprostatic adipose tissue biology. Third, the study did not directly evaluate AR expression, AR transcriptional activity, PSA levels, androgen-responsive genes, p-AKT, p-ERK, VEGFR, CCR2 expression, or NF-κB signaling in tumor tissues. Fourth, the xenograft model used here relies on immunodeficient SCID mice and therefore does not fully capture obesity-associated immune remodeling, immune cell recruitment, macrophage and T-cell interactions, antigen-specific antitumor immunity, or immune checkpoint regulation [46,47]. These limitations should be addressed in future studies using more comprehensive metabolic assessment, primary human adipocyte or periprostatic adipose tissue models, pathway-specific molecular assays, immunocompetent or humanized models, patient-derived models, and clinical biospecimens.

Finally, the translational feasibility of EGCG, GT, and arctigenin requires careful consideration. EGCG and arctigenin have known challenges related to absorption, metabolism, systemic exposure, tissue distribution, formulation, and clinically achievable concentrations. Therefore, the concentrations used in vitro and the exposures achieved in preclinical animal models should not be assumed to correspond directly to clinically achievable levels in humans. Future translational studies will need to evaluate pharmacokinetics, tissue bioavailability, safety, formulation strategies, and clinically relevant exposure levels before therapeutic relevance can be assessed.

## 5. Conclusions

Our findings suggest that HFD-associated metabolic and inflammatory alterations, adipocyte-associated interactions, proliferation, angiogenesis-related changes, and stress/apoptosis-related signaling features are linked within a tumor-supportive framework in preclinical prostate cancer models. Combined pharmacologic/nutritional modulation with GT, arctigenin, and RS 504393 was associated with reduced tumor growth and altered tumor-associated signaling features under HFD conditions. These results support a working model in which obesity-associated tumor support is network-influenced and may require further multi-pathway investigation, while additional mechanistic, immune-competent, metabolic, pharmacokinetic, and translational validation remains necessary.

## Data Availability

The original contributions presented in this study are included in the article/Supplementary Materials. Further inquiries can be directed to the corresponding authors.

## Supplementary Materials

The following supporting information can be downloaded at: https://www.mdpi.com/article/10.3390/biom16070969/s1, Figure S1. Body weight changes during the in vivo treatment period. Body weights of LAPC-4 xenograft-bearing mice maintained under LFD or HFD conditions and treated with GT, Arc, GT + Arc, RS 504393, GT + Arc + RS 504393, or vehicle control were monitored weekly during the 6-week treatment period. Data are presented as mean ± SD. HFD-fed mice showed an increased body weight trend compared with LFD control mice, while no excessive body weight loss was observed in the treatment groups compared with the HFD control group. LFD, low-fat diet; HFD, high-fat diet; GT, green tea; Arc, arctigenin; RS, RS 504393.
