# Supplementary material for: Combined Pharmacologic and Nutritional Modulation of High-Fat Diet-Associated Tumor-Supportive Features in Prostate Cancer Models
Source: Biomolecules. 2026 Jul 1;16(7):969. doi: 10.3390/biom16070969 (PMC13406970; doi:10.3390/biom16070969)
Supplement: Supplementary file 1 [file biomolecules-16-00969-s001.zip › biomolecules-4346786-supplementary.pdf]

Supplementary Figure S1

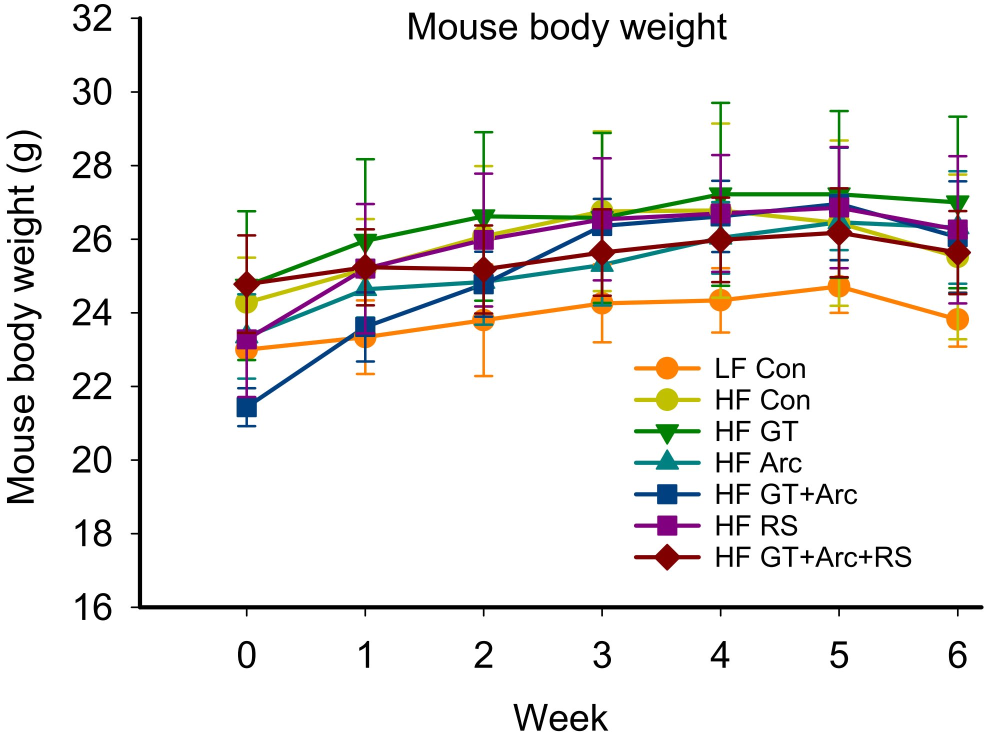

Supplementary Figure S1. Body weight changes during the in vivo treatment period. Body weights of LAPC-4 xenograft-bearing mice maintained under LFD or HFD conditions and treated with GT, Arc, GT + Arc, RS 504393, GT + Arc + RS 504393, or vehicle control were monitored weekly during the 6-week treatment period. Data are presented as mean ± SD. HFD-fed mice showed an increased body weight trend compared with LFD control mice, while no excessive body weight loss was observed in the treatment groups compared with the HFD control group. LFD, low-fat diet; HFD, high-fat diet; GT, green tea; Arc, arctigenin; RS, RS 504393.
